# Health-related quality of life in patients with primary brain tumors during and three months after treatment with proton beam therapy

**DOI:** 10.1016/j.tipsro.2021.01.004

**Published:** 2021-02-12

**Authors:** Ulrica Langegård, Per Fransson, Thomas Bjork-Eriksson, Birgitta Johansson, Emma Ohlsson-Nevo, Katarina Sjövall, Karin Ahlberg

**Affiliations:** a Institute of Health and Care Sciences, Sahlgrenska Academy, University of Gothenburg, Sweden; b Department of Nursing, Umeå University, Sweden; c Department of Cancer Centrum, Norrlands University Hospital, Umeå, Sweden; d Department of Oncology, Institute of Clinical Sciences, Sahlgrenska Academy at the University of Gothenburg, Sweden; e Regional Cancer Centre West, Western Sweden Healthcare Region, Gothenburg, Sweden; f Department of Immunology, Genetics and Pathology, Section of Oncology, Uppsala University, Uppsala, Sweden; g Department of Surgery, Faculty of Medicine and Health, Örebro University, Örebro, Sweden; h Department of Health and Society, Kristianstad University, Kristianstad, Sweden

**Keywords:** Proton beam therapy, Radiotherapy, Primary brain tumor, Symptom, Health related quality of life

## Abstract

**Background:**

Proton beam therapy (PBT) is increasingly administered to patients with primary brain tumors. Benefits of new treatments must be weighed against side effects and possible deterioration in health-related quality of life (HRQoL). The aim of this study was to describe and compare HRQoL, including acute symptom experiences and associated factors, in patients with malignant and benign brain tumors treated with PBT.

**Materials and Methods:**

Adult PBT-treated patients with primary brain tumors (n = 266) were studied. HRQoL was assessed with EORTC QLQ-C30, QLQ-BN20, HADS, ISI and MFI before, during and three months after treatment. Associations with demographic and medical factors were explored.

**Results:**

Between baseline and three months post-treatment: HRQoL decreased significantly in the global health/QOL domains physical functioning, role functioning and cognitive functioning in the malignant group, global health/QOL and physical functioning decreased significantly in the benign group, more comorbidity was significantly associated with increased motor dysfunction, leg weakness, headache and future uncertainty. Fatigue and depression were the most frequent symptoms in both groups. Independent predictors of risk factor recognition were age, sex, chemotherapy, comorbidity and education level.

**Discussion:**

Global health/QOL in patient with brain tumors is very complex and multidimensional. Symptoms are interrelated and related to patient, tumor and treatment factors. It is important to identify aspects of HRQoL that may be affected by treatment. These include both benefits, expected to improve HRQoL, and negative changes such as symptom experience and influencing factors. Evidence-based guidelines are needed for symptom management, and for high quality of care for patients experiencing low PBT-related HRQoL.

## Highlights

• Acute symptoms were common after proton beam therapy among patients with brain tumors.
• HRQoL was equally distributed among patients with malignant and benign brain tumors three months after proton beam therapy.
• Clinically relevant changes were found in patients with malignant and benign brain tumors treated with proton beam therapy.

## Introduction

Benefits of existing and new treatments must be weighed against side effects and possible deterioration in health-related quality of life (HRQoL) [1]. While conventional radiotherapy with photons (XRT) has been administered for years, proton beam therapy (PBT) is increasingly being administered to patients with primary brain tumors [2]. PBT offers the possibility to reduce non-desirable radiation doses to healthy brain tissue, mainly due to the advantageous physical properties of protons [3].

Primary brain tumors are relatively infrequent and are classified as malignant or benign, according to the World Health Organization classification [4]. About 238,000 patients are annually diagnosed with malign brain tumor worldwide [5]. In Sweden, approximately 1400 patients are diagnosed annually, and approximately 50% have malignant tumors [6].

Malignant gliomas are heterogeneous, highly invasive primary brain tumors and are managed by surgical removal of as much of the tumor bulk as is considered safe, followed by fractionated radiotherapy (RT; typically 60 Gy in 30–35 fractions), and concurrent chemotherapy, which is given continuously for at least an additional six months after cessation of RT [7]. Asymptomatic benign brain tumors can be followed up frequently until they become symptomatic, and then surgically resected and treated with adjuvant radiotherapy (RT) [8]. Total surgical resection of benign brain tumors is generally the treatment of choice since it results in long-term disease-free survival in most patients. RT does not generally eradicate a benign tumor but does eliminate its capability for growth [9]. The follow-up for patients in this category may be long, even as long as for tumors that are malignant. The effect on HRQoL related to the symptoms may be just as severe, and the tumor may be incurable in some cases with a benign diagnosis [8].

Primary symptoms in patients with brain tumors are headache, anorexia, nausea, vomiting, seizures, longer nocturnal sleeping and daytime drowsiness [10]. Fatigue, double vision, neurological deficits, cognitive impairment and insomnia are also common [11], [12]. Furthermore, depression is common, but often under-recognized and untreated, complication in patients with brain tumors [13]. These symptoms may impact the patients HRQoL including functioning’s and well-being [14], [15].

Maintenance or improvement of HRQoL, including symptom experience, is an important treatment goal [1], [16], [17]. More research is needed among patient reported outcomes in patients with primary brain tumors receiving PBT. The aim of this study was therefore to describe and compare HRQoL, including acute symptom experiences and associated factors in patients with malignant and benign brain tumors treated with PBT.

## Methods

### Study design

This study is part of ProtonCare, a larger multicenter project assessing the role of proton treatment compared to other modern photon based radiotherapy techniques. The ultimate purpose of ProtonCare is to investigate patient-reported variables, e.g. short- and long-term symptoms and HRQoL in patients receiving PBT. This study has a quantitative, longitudinal and descriptive design.

### Setting and treatments

The Skandion Clinic is situated in Uppsala, Sweden, and managed jointly by the seven Swedish regions hosting university hospital RT departments (local departments). Patients with primary brain tumors, eligible for PBT, are evaluated during bi-weekly video conferences between the Skandion Clinic and these RT departments. Treatment plans and immobilization devices for PBT patients are transferred to the Skandion Clinic, which is responsible for treatment and for clinical evaluations during treatment. Patients are subsequently referred to their local department for long-term follow-up.

### Patients and procedure

A consecutive sample of 301 patients referred to PBT at the Skandion Clinic between August 2015 and October 2018 were invited to participate in the study. These patients were part of a multi-center prospective PBT protocol that included adult patients with primary central nervous system tumors [18]. As in the PRO-CNS protocol [18], we included patients with both malignant (Low grade gliomas - grade I-II and anaplastic glioma grade III with Loss of Heterozygosity (LOH) 1p/19q) and benign brain tumors where surgery was not the only treatment of choice. All included patients with benign tumors had non-resectable tumors and substantial tumor volumes, and repeated computed tomography or magnetic resonance imaging had revealed continuous tumor growth. The benign tumors thus constituted a life-threatening condition requiring the same treatment as malignant tumors. Target doses, gross tumor volumes and planning target volumes as well as radiation techniques were comparable with those for malignant brain tumors. However, clinical target volumes varied compared to the malignant tumors. Even in the group of malignant tumors, the clinical target volumes differed between 10 and 20 mm for low-grade and high-grade tumors, respectively. Inclusion criteria were age ≥18 years, primary brain tumor, scheduled for PBT and able to communicate in Swedish. Study information was provided by the first author (UL) by telephone. Written information, including the voluntary nature of participation, confidentiality and freedom to withdraw from the study, was sent to interested patients by mail. All participants provided written informed consent before data collection started. The study was approved by the Research Ethics Committee in Gothenburg, Sweden (permit number Dnr:433–15).

### Data collection

#### Medical and demographic data

Medical data were collected from medical records. Patient characteristics, i.e. age, sex, occupational status, education and comorbidities, were collected with project-specific questionnaires.

### Questionnaires

#### Comorbidity

Comorbidities were assessed at treatment start with the Self-Administered Comorbidity Questionnaire (SCQ), originally developed by Sangha, Stucki, Liang, Fossel and Katz [19]. The SCQ asks “Do you have any of the following problems?” and lists 15 common medical problems. For each problem, participants were asked “Do you receive treatment?” as a proxy for disease severity, followed by the question, “Does it limit your daily activities?” Participants scored a maximum of three points for each condition (Supplementary File 1).

#### Multidimensional fatigue inventory

Fatigue was measured with the Multidimensional Fatigue Inventory (MFI-20) [20], [21]. This questionnaire consists of 20 items that assess five dimensions of fatigue based on different modes of expression: general fatigue; physical fatigue; reduced activities; lack of motivation; and mental fatigue. Each dimension contains four items, two indicating and two contraindicating fatigue. The response ranges from agreement with the accompanying statement (“Yes, that is true”) to disagreement (“No, that is not true”). A total score is calculated for each scale by summation of the individual item scores, that range from 4 to 20 [20].

#### Hospital anxiety and depression scale

The Hospital Anxiety and Depression Scale (HADS) [22] is a 14-item screening questionnaire, with seven items respectively relating to anxiety (HADS-A) and depression (HADS-D). Ratings are made on a four-point scale with scores ranging from 0 (no symptoms of depression or anxiety) to 21 (numerous and severe symptoms) for each item. HADS scores are classified as follows: 0–7 = non-cases, 8–10 = doubtful cases and 11–21 = cases [22].

#### Insomnia severity index

Sleep disturbance was measured with the seven-item Insomnia Severity Index (ISI) [23]. The ISI uses a five-point Likert scale to rate difficulty with sleep onset, sleep maintenance and early morning awakening, as well as interference with daytime functioning, how noticeable sleep problems are to others, distress caused by problematic sleep and overall sleep satisfaction. Total scores range from 0 to 28, with higher scores indicating greater severity.

#### EORTC QLQ-C30 and QLQ-BN20

HRQoL was measured with the European Organization for Research and Treatment of Cancer (EORTC) QLQ-C30 [17] and QLQ-BN20 [24] questionnaires. The QLQ-C30 contains five functioning scales, three symptom scales, six single items and global health status/quality of life (QOL) scale. The instrument determines which score change magnitude corresponds to change defined by the patient as significant. All scales and single items are transformed into scores ranging from 0 to 100. For functional scales, global QOL and summary score, a higher score suggests a better level of functioning, while a higher score suggests more severe problems when it comes to symptoms [25]. The QLQ-BN20 questionnaire is a brain-specific module to be used in conjunction with the QLQ-C30, containing 20 items grouped into four multi-item scales (future uncertainty, visual disorder, motor dysfunction and communication deficit) and seven single items. Data were processed according to the EORTC QLQ-C30 manual [25].

#### Procedure

All questionnaires were to be completed at start of treatment, three weeks after treatment start, at end of treatment and at one and three months after end of treatment. The participants could choose to respond online or on paper, as previous research suggests little discrepancy in reliability between these formats [26]. A link to the online questionnaires was e-mailed to participants at each assessment, followed by a reminder after one week, if necessary. Patients choosing paper received the questionnaires and a pre-paid envelope at the RT department, or by mail after treatment ended. A reminder was sent if questionnaires were not returned within one week.

### Statistical analysis

#### Descriptive analysis

Numbers and percentages are presented for categorical variables and means and standard deviations (SD) are presented for continuous variables. For comparison between groups, the Mantel-Haenszel Chi Square test was used for ordered categorical variables and the Mann-Whitney *U* test for continuous variables. The Wilcoxon Signed Rank test was used to analyze changes over time within the treatment group. These non-parametric methods were chosen because the majority of the data analyzed were skewed, and missing values were imputed using the last value carried forward method [27], shown in Table 3. Additionally, changes in clinical significance over time were assessed in QLQ-C30, according to Osoba et al. [28] and in QLQ-BN20 according to Wong et al. [29] based on the observed percentages with decrease or increase of at least five points on the respective subscale or for single items at three-month follow-up. Further, the clinical relevance was calculated in HADS-A and HADS-D with decrease or increase of at least 1.68 and 1.60 respectively according to Puhan et al. [30], MFI 2.0 points according to Pursell et al. [31] and ISI with a 6-point score according to Yang et al. [32].

#### Regression analysis

Linear regression analysis was applied to analyze how demographic and medical data were associated with change in HRQoL from baseline to three months after treatment. The dependent QLQ-C30 variables were global health/QOL and physical, role, emotional, cognitive and social functioning. The selected symptoms were fatigue, nausea, pain and insomnia. In addition to all dimensions in the MFI, HAD and ISI, the scales future uncertainty, visual disorder, motor dysfunction and communication deficit, well-known brain tumor symptoms, were chosen from the QLQ-BN20. Variables that were significant in the univariable analysis (p < 0.1) were entered into a forward stepwise multivariable regression model. Beta estimates with 95% confidence intervals, p-values, and r^2^ were calculated. Comorbidity incidence was low. Therefore, in order to include this variable, patients were dichotomized based on SCQ cut-offs: 0–3 or >4 points.

Statistical analyses were performed using the SAS system, version 9.4. Reported p-values are two-tailed, and *p* < 0.05 was considered statistically significant.

## Results

A total of 266 of 301 (88%) patients diagnosed with primary brain tumors, and treated with PBT (malignant tumors, n = 159; benign tumors, n = 107), agreed to participate. All included patients with benign tumors had non-resectable tumors. The majority of the patients were in good performance status ECOG 0–1 and KPS 80–100%. Characteristics of the study population are shown in Table 1.

**Table 1:** Demographic and clinical characteristics of patients in total (n = 266) with primary brain tumors undergoing proton beam therapy.

Parameters	Malign tumor n (%)	Benign tumor n (%)
**Diagnosis**	159 (60)	107 (40)
C 70: Malignant tumor in CNS meningium	19 (12)	
C 71: Malignant tumor in the brain	140 (88)	
D 32: Benign tumor in CNS meningium		85 (79)
D 33: Benign tumor in in the brain		22 (21)
**Sex**		
Women	73 (46)	67 (63)
Men	86 (54)	40 (37)
**Treatment**		
Total dose, Gy [median, (range)]	52 (34–60)	52 (45–54)
Number of fractions (min, max)	10, 33	23–33
**Diagnose**		
Comorbidity, SCQ category*		
<4	133 (84)	98 (92)
>4	20 (13)	9 (8)
Missing	6 (3)	
**Age, years**		
Mean	44	56
SD	12,4	13,4
Min	19	24
Max	75	80
**Marital status**		
Married	110 (69)	74 (69)
Single	48 (30)	33 (31)
Missing	1 (1)	–
**Education**		
Elementary	12 (7)	14 (13)
Secondary	66 (42)	56 (52)
University	77 (48)	36 (34)
Missing	4 (3)	1 (1)
**Questionnaire format**		
Paper	87 (55)	77 (72)
Digital	72 (45)	30 (28)

*SCQ **=** Self-Administered Comorbidity Questionnaire. Participants scored a maximum of 3 points for each condition: one for existence of the problem, one for treatment and one for limited activities (maximum 45 points).

### HRQoL and symptoms in the malignant tumor group

Statistically significant deteriorations was found between baseline and three months post-treatment, and the worst deteriorations were in global health/QOL, fatigue, appetite loss, constipation, drowsiness and hair loss. Improvements between baseline and three months post-treatment were found in insomnia (Table 2). Changes on the HADS between baseline and three months post-treatment shows that the HADS-A scores underwent significant improvements, the ISI scores were essentially unchanged, while scores in all MFI dimensions significantly deteriorated (Table 2).

**Table 2:** Responses to the QLQC30, BN20, HAD, ISI and MFI (mean, SD) from patients with malignant and benign brain tumors, during treatment with proton beam therapy, at follow up one and three months after treatment.

**Variable**	**Visit**														
	**Baseline**			**MID**			**END**			**1 month**			**3 months**		
	**Malign**	**Benign**	**p-value**	**Malign**	**Benign**	**p-value**	**Malign**	**Benign**	**p-value**	**Malign**	**Benign**	**p-value**	**Malign**	**Benign**	**p-value**
	n = 159	n = 107		n = 159	n = 107		n = 159	n = 107		n = 159	n = 107		n = 159	n = 107	
**Global health status**															
**mean (SD)**															
	65.9 (20.0)	70.4 (18.6)	0.089	63.3 (20.0)	65.7 (20.5)	0.41	59.2 (20.9)	60.2 (22.8)	0.62	58.5 (23.7)	62.5 (24.9)	0.19	59.6 (23.1)	64.5 (22.1)	0.11
**Change** **mean (SD)**				−2.62 (13.16) p = 0.0086	−4.67 (17.44) p = 0.020	0.54	−6.66 (16.61) p < 0.0001	−10.2 (19.4) p < 0.0001	0.32	−7.34 (19.85) p < 0.0001	−7.94 (21.67) p = 0.0005	0.80	−6.29 (19.93) p < 0.0001	−5.92 (20.12) p = 0.0045	0.50
**Improved, n (%)**				42 (26.4%)	24 (22.4%)		32 (20.1%)	21 (19.6%)		34 (21.4%)	27 (25.2%)		36 (22.6%)	28 (26.2%)	
**Unchanged, n (%)**				51 (32.1%)	37 (34.6%)		37 (23.3%)	25 (23.4%)		41 (25.8%)	24 (22.4%)		41 (25.8%)	30 (28.0%)	
**Impaired, n (%)**				66 (41.5%)	46 (43.0%)		90 (56.6%)	61 (57.0%)		84 (52.8%)	56 (52.3%)		82 (51.6%)	49 (45.8%)	
**Physical functioning**															
**mean (SD)**	84.9 (17.9)	85.0 (18.1)	0.99	84.4 (20.4)	82.9 (19.8)	0.32	83.6 (20.2)	81.8 (19.6)	0.23	81.5 (21.9)	80.8 (21.0)	0.51	80.5 (22.1)	82.6 (17.7)	0.96
**Change** **mean (SD)**				−0.503 (11.214) p = 0.93	−2.12 (11.53) p = 0.081	0.14	−1.38 (11.43) p = 0.15	−3.18 (13.62) p = 0.016	0.19	−3.44 (14.13) p = 0.0097	−4.21 (14.26) p = 0.0042	0.53	−4.40 (14.29) p = 0.0002	−2.37 (11.77) p = 0.046	0.46
**Improved, n (%)**				44 (27.7%)	22 (20.6%)		37 (23.3%)	23 (21.5%)		41 (25.8%)	22 (20.6%)		38 (23.9%)	24 (22.4%)	
**Unchanged, n (%)**				73 (45.9%)	50 (46.7%)		72 (45.3%)	39 (36.4%)		61 (38.4%)	46 (43.0%)		55 (34.6%)	43 (40.2%)	
**Impaired, n (%)**				42 (26.4%)	35 (32.7%)		50 (31.4%)	45 (42.1%)		57 (35.8%)	39 (36.4%)		66 (41.5%)	40 (37.4%)	
**Role functioning**															
**mean (SD)**	61.6 (34.7)	68.5 (33.9)	0.073	61.1 (34.3)	64.5 (34.6)	0.35	53.8 (35.3)	61.4 (32.9)	0.090	56.3 (33.2)	63.1 (31.9)	0.10	56.7 (32.5)	68.5 (30.6)	0.0030
**Change** **mean (SD)**				−0.524 (25.603) p = 0.83	−4.05 (31.13) p = 0.15	0.23	−7.86 (28.55) p < 0.0001	−7.17 (36.21) p = 0.023	0.83	−5.35 (28.27) p = 0.0068	−5.45 (34.32) p = 0.056	0.62	−4.93 (30.44) p = 0.019	0.000 (34.110) p = 0.72	0.37
**Improved, n (%)**				42 (26.4%)	21 (19.6%)		34 (21.4%)	23 (21.5%)		40 (25.2%)	23 (21.5%)		41 (25.8%)	30 (28.0%)	
**Unchanged, n (%)**				73 (45.9%)	52 (48.6%)		57 (35.8%)	42 (39.3%)		58 (36.5%)	41 (38.3%)		55 (34.6%)	39 (36.4%)	
**Impaired, n (%)**				44 (27.7%)	34 (31.8%)		68 (42.8%)	42 (39.3%)		61 (38.4%)	43 (40.2%)		63 (39.6%)	38 (35.5%)	
**Emotional functioning**															
**mean (SD)**	73.9 (21.7)	76.6 (21.7)	0.23	81.1 (19.8)	83.8 (21.5)	0.062	80.7 (20.5)	82.6 (23.9)	0.10	78.2 (21.8)	80.5 (23.6)	0.15	76.8 (21.6)	78.8 (23.6)	0.18
**Change** **mean (SD)**				7.18 (17.33) p < 0.0001	7.17 (16.92) p < 0.0001	0.68	6.81 (19.42) p < 0.0001	6.00 (17.35) p < 0.0001	0.80	4.30 (20.54) p = 0.0034	3.89 (18.75) p = 0.017	0.89	2.88 (21.26) p = 0.070	2.18 (17.82) p = 0.13	0.99
**Improved, n (%)**				81 (50.9%)	55 (51.4%)		80 (50.3%)	58 (54.2%)		75 (47.2%)	52 (48.6%)		74 (46.5%)	48 (44.9%)	
**Unchanged, n (%)**				49 (30.8%)	36 (33.6%)		49 (30.8%)	30 (28.0%)		40 (25.2%)	27 (25.2%)		35 (22.0%)	30 (28.0%)	
**Impaired, n (%)**				29 (18.2%)	16 (15.0%)		30 (18.9%)	19 (17.8%)		44 (27.7%)	28 (26.2%)		50 (31.4%)	29 (27.1%)	
**Cognitive functioning**															
**mean (SD)**	78.3 (22.5)	78.8 (22.3)	0.86	79.7 (21.9)	80.5 (21.7)	0.72	75.8 (24.3)	76.9 (23.6)	0.69	74.9 (25.9)	75.2 (23.0)	0.75	72.4 (25.2)	76.5 (22.5)	0.25
**Change** **mean (SD)**				1.36 (17.88) p = 0.38	1.71 (16.97) p = 0.41	0.81	−2.52 (20.47) p = 0.091	−1.87 (19.20) p = 0.26	1.00	−3.35 (21.77) p = 0.084	−3.58 (20.99) p = 0.072	0.51	−5.87 (20.64) p = 0.0004	−2.34 (18.38) p = 0.18	0.17
**Improved, n (%)**				42 (26.4%)	31 (29.0%)		41 (25.8%)	26 (24.3%)		42 (26.4%)	22 (20.6%)		33 (20.8%)	25 (23.4%)	
**Unchanged, n (%)**				82 (51.6%)	53 (49.5%)		71 (44.7%)	49 (45.8%)		64 (40.3%)	46 (43.0%)		63 (39.6%)	50 (46.7%)	
**Impaired, n (%)**				35 (22.0%)	23 (21.5%)		47 (29.6%)	32 (29.9%)		53 (33.3%)	39 (36.4%)		63 (39.6%)	32 (29.9%)	
**Social functioning**															
**mean (SD)**	70.2 (28.5)	78.2 (26.3)	0.014	68.4 (29.7)	76.0 (26.2)	0.039	66.6 (28.4)	76.5 (25.4)	0.0031	69.4 (26.7)	75.4 (26.0)	0.051	67.7 (28.4)	80.1 (24.6)	0.0003
**Change** **mean (SD)**				−1.78 (24.28) p = 0.42	−2.18 (19.97) p = 0.22	0.67	−3.67 (26.16) p = 0.10	−1.71 (21.59) p = 0.24	0.63	−0.839 (27.863) p = 0.64	−2.80 (24.39) p = 0.18	0.74	−2.52 (28.51) p = 0.13	1.87 (21.76) p = 0.38	0.14
**Improved, n (%)**				44 (27.7%)	25 (23.4%)		44 (27.7%)	24 (22.4%)		46 (28.9%)	27 (25.2%)		49 (30.8%)	33 (30.8%)	
**Unchanged, n (%)**				69 (43.4%)	51 (47.7%)		54 (34.0%)	52 (48.6%)		61 (38.4%)	48 (44.9%)		52 (32.7%)	50 (46.7%)	
**Impaired, n (%)**				46 (28.9%)	31 (29.0%)		61 (38.4%)	31 (29.0%)		52 (32.7%)	32 (29.9%)		58 (36.5%)	24 (22.4%)	
**Fatigue**															
**mean (SD)**	33.2 (24.8)	30.4 (24.2)	0.39	35.3 (24.6)	37.0 (26.4)	0.70	41.4 (26.3)	42.7 (27.1)	0.83	42.8 (28.0)	41.8 (27.5)	0.78	42.1 (28.3)	37.9 (25.7)	0.23
**Change** **mean (SD)**				2.10 (16.31) p = 0.12	6.54 (19.48) p = 0.0005	0.044	8.25 (19.58) p < 0.0001	12.3 (22.0) p < 0.0001	0.14	9.64 (22.12) p < 0.0001	11.4 (22.9) p < 0.0001	0.70	8.91 (22.22) p < 0.0001	7.48 (22.26) p = 0.0008	0.58
**Improved, n (%)**				43 (27.0%)	22 (20.6%)		33 (20.8%)	16 (15.0%)		35 (22.0%)	21 (19.6%)		37 (23.3%)	27 (25.2%)	
**Unchanged, n (%)**				69 (43.4%)	40 (37.4%)		51 (32.1%)	35 (32.7%)		38 (23.9%)	29 (27.1%)		37 (23.3%)	26 (24.3%)	
**Impaired, n (%)**				47 (29.6%)	45 (42.1%)		75 (47.2%)	56 (52.3%)		86 (54.1%)	57 (53.3%)		85 (53.5%)	54 (50.5%)	
**Nausea**															
**mean (SD)**	6.29 (16.53)	3.58 (12.96)	0.040	8.39 (16.65)	7.79 (16.24)	0.55	7.97 (15.10)	7.63 (14.89)	0.69	10.2 (19.1)	6.07 (12.41)	0.13	11.2 (18.0)	7.32 (16.54)	0.018
**Change** **mean (SD)**				2.10 (13.75) p = 0.056	4.21 (12.35) p = 0.0002	0.23	1.68 (16.37) p = 0.13	4.05 (13.51) p = 0.0007	0.16	3.88 (19.05) p = 0.011	2.49 (10.93) p = 0.0097	0.96	4.93 (19.17) p = 0.0011	3.74 (15.41) p = 0.010	0.41
**Improved, n (%)**				18 (11.3%)	4 (3.7%)		23 (14.5%)	5 (4.7%)		22 (13.8%)	6 (5.6%)		18 (11.3%)	7 (6.5%)	
**Unchanged, n (%)**				108(67.9%)	80 (74.8%)		98 (61.6%)	76 (71.0%)		99 (62.3%)	82 (76.6%)		96 (60.4%)	80 (74.8%)	
**Impaired, n (%)**				33 (20.8%)	23 (21.5%)		38 (23.9%)	26 (24.3%)		38 (23.9%)	19 (17.8%)		45 (28.3%)	20 (18.7%)	
**Pain**															
**mean (SD)**	14.3 (22.3)	15.4 (23.6)	0.82	16.7 (22.1)	19.8 (24.8)	0.40	17.3 (23.0)	24.8 (28.5)	0.038	17.9 (25.0)	22.1 (28.1)	0.34	16.5 (23.9)	19.8 (26.7)	0.34
**Change** **mean (SD)**				2.41 (18.64) p = 0.15	4.36 (19.74) p = 0.0074	0.19	3.04 (18.74) p = 0.046	9.35 (26.12) p < 0.0001	0.016	3.67 (20.25) p = 0.055	6.70 (23.89) p = 0.0018	0.16	2.20 (19.50) p = 0.27	4.36 (23.05) p = 0.061	0.32
**Improved, n (%)**				29 (18.2%)	15 (14.0%)		29 (18.2%)	14 (13.1%)		28 (17.6%)	17 (15.9%)		30 (18.9%)	21 (19.6%)	
**Unchanged, n (%)**				88 (55.3%)	57 (53.3%)		85 (53.5%)	49 (45.8%)		87 (54.7%)	52 (48.6%)		92 (57.9%)	52 (48.6%)	
**Impaired, n (%)**				42 (26.4%)	35 (32.7%)		45 (28.3%)	44 (41.1%)		44 (27.7%)	38 (35.5%)		37 (23.3%)	34 (31.8%)	
**Dyspnea**															
**mean (SD)**	22.0 (26.5)	14.5 (24.4)	0.0064	23.1 (28.1)	16.5 (25.2)	0.035	23.9 (26.0)	22.7 (27.7)	0.52	29.8 (28.0)	25.5 (30.9)	0.099	25.8 (27.3)	24.0 (28.9)	0.43
**Change** **mean (SD)**				1.05 (24.43) p = 0.56	1.89 (21.00) p = 0.36	0.69	1.89 (20.96) p = 0.26	8.18 (22.92) p = 0.0002	0.030	7.76 (24.65) p < 0.0001	11.3 (26.0) p < 0.0001	0.36	3.77 (27.04) p = 0.058	9.43 (25.51) p = 0.0001	0.13
**Improved, n (%)**				30 (18.9%)	13 (12.3%)		23 (14.5%)	10 (9.4%)		21 (13.2%)	10 (9.4%)		30 (18.9%)	12 (11.3%)	
**Unchanged, n (%)**				98 (61.6%)	76 (71.7%)		105(66.0%)	64 (60.4%)		88 (55.3%)	60 (56.6%)		85 (53.5%)	60 (56.6%)	
**Impaired, n (%)**				31 (19.5%)	17 (16.0%)		31 (19.5%)	32 (30.2%)		50 (31.4%)	36 (34.0%)		44 (27.7%)	34 (32.1%)	
**Insomnia**															
**mean (SD)**	27.0 (28.6)	21.5 (29.8)	0.051	25.8 (28.5)	28.7 (30.9)	0.53	28.9 (30.0)	31.2 (34.0)	0.84	26.8 (30.6)	24.9 (30.7)	0.54	21.6 (27.3)	21.5 (30.1)	0.63
**Change** **mean (SD)**				−1.26 (24.84) p = 0.57	7.17 (26.31) p = 0.0050	0.023	1.89 (27.11) p = 0.41	9.66 (31.06) p = 0.0015	0.072	−0.210 (29.170) p = 0.85	3.43 (26.28) p = 0.18	0.17	−5.45 (30.67) p = 0.016	0.000 (28.225) p = 0.95	0.18
**Improved, n (%)**				34 (21.4%)	13 (12.1%)		34 (21.4%)	15 (14.0%)		39 (24.5%)	20 (18.7%)		47 (29.6%)	23 (21.5%)	
**Unchanged, n (%)**				94 (59.1%)	65 (60.7%)		86 (54.1%)	59 (55.1%)		87 (54.7%)	58 (54.2%)		85 (53.5%)	65 (60.7%)	
**Impaired, n (%)**				31 (19.5%)	29 (27.1%)		39 (24.5%)	33 (30.8%)		33 (20.8%)	29 (27.1%)		27 (17.0%)	19 (17.8%)	
**Appetite**															
**mean (SD)**	9.85 (21.40)	8.41 (18.37)	0.74	14.5 (23.6)	14.3 (24.7)	0.85	14.7 (25.6)	15.6 (23.5)	0.40	17.6 (25.7)	10.9 (21.9)	0.014	19.7 (26.3)	15.0 (24.8)	0.83
**Change** **mean (SD)**				4.61 (22.02) p = 0.0098	5.92 (21.38) p = 0.0034	0.71	4.82 (27.00) p = 0.023	7.17 (22.44) p = 0.0008	0.38	7.76 (27.86) p = 0.0003	2.49 (20.83) p = 0.25	0.065	9.85 (26.93) p < 0.0001	6.54 (24.42) p = 0.0058	0.17
**Improved, n (%)**				12 (7.5%)	8 (7.5%)		18 (11.3%)	7 (6.5%)		16 (10.1%)	11 (10.3%)		11 (6.9%)	11 (10.3%)	
**Unchanged, n (%)**				117(73.6%)	76 (71.0%)		106(66.7%)	74 (69.2%)		99 (62.3%)	79 (73.8%)		97 (61.0%)	69 (64.5%)	
**Impaired, n (%)**				30 (18.9%)	23 (21.5%)		35 (22.0%)	26 (24.3%)		44 (27.7%)	17 (15.9%)		51 (32.1%)	27 (25.2%)	
**Constipation**															
**mean (SD)**	6.16 (17.22)	6.54 (19.66)	0.82	9.64 (19.97)	8.10 (19.34)	0.42	10.9 (21.4)	11.5 (24.3)	0.87	8.60 (19.20)	5.61 (14.10)	0.26	14.3 (22.6)	10.6 (21.3)	0.11
**Change** **mean (SD)**				3.40 (20.04) p = 0.022	1.56 (18.53) p = 0.39	0.25	4.88 (22.27) p = 0.0030	4.98 (23.70) p = 0.034	0.91	2.34 (23.30) p = 0.23	−0.935 (16.161) p = 0.47	0.27	7.86 (24.79) p < 0.0001	4.05 (23.22) p = 0.11	0.24
**Improved, n (%)**				10 (6.4%)	9 (8.4%)		10 (6.4%)	9 (8.4%)		13 (8.3%)	8 (7.5%)		10 (6.4%)	7 (6.5%)	
**Unchanged, n (%)**				122(77.7%)	86 (80.4%)		121(77.1%)	79 (73.8%)		122(77.7%)	91 (85.0%)		107(68.2%)	80 (74.8%)	
**Impaired, n (%)**				25 (15.9%)	12 (11.2%)		26 (16.6%)	19 (17.8%)		22 (14.0%)	8 (7.5%)		40 (25.5%)	20 (18.7%)	
**Diarrhoea**															
**mean (SD)**	9.85 (19.33)	3.43 (10.17)	0.0029	8.81 (18.92)	5.61 (14.83)	0.13	8.60 (19.56)	6.54 (16.15)	0.44	9.64 (20.66)	7.79 (18.07)	0.50	8.81 (17.77)	8.72 (19.06)	0.75
**Change** **mean (SD)**				−1.05 (17.36) p = 0.45	2.18 (16.68) p = 0.17	0.24	−1.26 (19.80) p = 0.45	3.12 (16.84) p = 0.065	0.062	−0.210 (19.666) p = 0.91	4.36 (20.00) p = 0.023	0.10	−1.05 (19.28) p = 0.57	5.30 (18.96) p = 0.0024	0.011
**Improved, n (%)**				19 (11.9%)	7 (6.5%)		24 (15.1%)	6 (5.6%)		21 (13.2%)	7 (6.5%)		22 (13.8%)	5 (4.7%)	
**Unchanged, n (%)**				125(78.6%)	89 (83.2%)		118(74.2%)	88 (82.2%)		119(74.8%)	84 (78.5%)		120(75.5%)	84 (78.5%)	
**Impaired, n (%)**				15 (9.4%)	11 (10.3%)		17 (10.7%)	13 (12.1%)		19 (11.9%)	16 (15.0%)		17 (10.7%)	18 (16.8%)	
**Financial difficulties**															
**mean (SD)**	25.4 (28.2)	16.5 (27.2)	0.0022	21.6 (27.6)	13.7 (24.6)	0.0061	23.3 (29.9)	17.4 (28.4)	0.048	20.8 (28.0)	18.4 (28.7)	0.32	22.9 (29.3)	14.6 (25.6)	0.011
**Change** **mean (SD)**				−3.77 (20.53) p = 0.022	−2.80 (17.80) p = 0.10	0.54	−2.10 (23.63) p = 0.28	0.935 (18.575) p = 0.63	0.18	−4.61 (25.57) p = 0.026	1.87 (21.39) p = 0.41	0.025	−2.52 (27.95) p = 0.24	−1.87 (22.82) p = 0.44	0.42
**Improved, n (%)**				30 (18.9%)	14 (13.1%)		33 (20.8%)	9 (8.4%)		37 (23.3%)	10 (9.3%)		36 (22.6%)	15 (14.0%)	
**Unchanged, n (%)**				114(71.7%)	85 (79.4%)		103(64.8%)	86 (80.4%)		101(63.5%)	81 (75.7%)		101(63.5%)	80 (74.8%)	
**Impaired, n (%)**				15 (9.4%)	8 (7.5%)		23 (14.5%)	12 (11.2%)		21 (13.2%)	16 (15.0%)		22 (13.8%)	12 (11.2%)	
**BN20 Future uncertainty**															
**mean (SD)**	24.8 (19.9)	18.6 (22.4)	0.0006	24.4 (19.5)	19.0 (22.2)	0.0016	23.9 (19.6)	19.9 (23.4)	0.0058	25.6 (20.8)	20.6 (22.2)	0.012	25.5 (20.6)	21.5 (24.5)	0.017
**Change** **mean (SD)**				−0.374 (18.903) p = 0.55	0.167 (16.916) p = 0.65	0.28	−0.908 (17.651) p = 0.54	1.08 (17.35) p = 0.26	0.25	0.837 (19.960) p = 0.61	1.75 (16.55) p = 0.24	0.63	0.730 (19.708) p = 0.65	2.67 (17.28) p = 0.096	0.27
**Improved, n (%)**				63 (40.4%)	28 (28.0%)		56 (35.9%)	25 (25.0%)		57 (36.5%)	29 (29.0%)		62 (39.7%)	29 (29.0%)	
**Unchanged, n (%)**				43 (27.6%)	39 (39.0%)		46 (29.5%)	37 (37.0%)		36 (23.1%)	30 (30.0%)		36 (23.1%)	32 (32.0%)	
**Impaired, n (%)**				50 (32.1%)	33 (33.0%)		54 (34.6%)	38 (38.0%)		63 (40.4%)	41 (41.0%)		58 (37.2%)	39 (39.0%)	
**BN20 Visual disorder**															
**mean (SD)**	9.90 (16.58)	13.9 (20.7)	0.15	11.9 (18.0)	14.2 (21.4)	0.72	11.8 (18.3)	16.8 (22.4)	0.051	13.2 (19.3)	16.1 (22.4)	0.42	12.5 (18.4)	16.4 (24.7)	0.19
**Change** **mean (SD)**				1.99 (15.84) p = 0.081	−0.111 (12.878) p = 0.92	0.42	1.85 (16.54) p = 0.15	2.56 (15.30) p = 0.13	0.68	3.28 (17.68) p = 0.023	1.89 (17.45) p = 0.51	0.48	2.56 (16.25) p = 0.035	2.22 (17.23) p = 0.19	0.68
**Improved, n (%)**				23 (14.7%)	19 (19.0%)		30 (19.2%)	18 (18.0%)		26 (16.7%)	19 (19.0%)		27 (17.3%)	21 (21.0%)	
**Unchanged, n (%)**				97 (62.2%)	59 (59.0%)		91 (58.3%)	57 (57.0%)		83 (53.2%)	54 (54.0%)		87 (55.8%)	53 (53.0%)	
**Impaired, n (%)**				36 (23.1%)	22 (22.0%)		35 (22.4%)	25 (25.0%)		47 (30.1%)	27 (27.0%)		42 (26.9%)	26 (26.0%)	
**BN20 Motor dysfunction**															
**mean (SD)**	11.6 (19.9)	8.39 (13.64)	0.77	12.3 (20.9)	10.3 (17.0)	0.87	12.7 (20.0)	11.9 (18.0)	0.87	14.5 (21.2)	13.4 (21.1)	0.69	14.2 (21.0)	13.5 (18.7)	0.92
**Change** **mean (SD)**				0.641 (15.263) p = 0.74	1.61 (14.13) p = 0.23	0.60	1.07 (12.98) p = 0.35	3.17 (14.74) p = 0.016	0.35	2.85 (12.93) p = 0.0066	4.72 (15.96) p = 0.0061	0.71	2.56 (13.58) p = 0.021	4.83 (13.83) p = 0.0006	0.26
**Improved, n (%)**				25 (16.0%)	16 (16.0%)		25 (16.0%)	14 (14.0%)		20 (12.8%)	13 (13.0%)		22 (14.1%)	15 (15.0%)	
**Unchanged, n (%)**				103(66.0%)	62 (62.0%)		94 (60.3%)	58 (58.0%)		90 (57.7%)	56 (56.0%)		91 (58.3%)	48 (48.0%)	
**Impaired, n (%)**				28 (17.9%)	22 (22.0%)		37 (23.7%)	28 (28.0%)		46 (29.5%)	31 (31.0%)		43 (27.6%)	37 (37.0%)	
**BN20 Communication deficit**															
**mean (SD)**	12.7 (18.7)	9.89 (17.29)	0.20	15.1 (22.0)	13.1 (20.6)	0.41	14.7 (19.7)	14.9 (20.7)	0.98	16.6 (19.1)	14.1 (19.1)	0.14	17.4 (21.1)	13.9 (18.9)	0.19
**Change** **mean (SD)**				2.42 (18.08) p = 0.26	2.78 (15.74) p = 0.061	0.63	2.07 (15.86) p = 0.17	4.67 (16.12) p = 0.0027	0.15	3.92 (15.86) p = 0.0016	3.78 (16.12) p = 0.016	0.63	4.70 (16.12) p = 0.0007	3.67 (14.99) p = 0.0082	0.97
**Improved, n (%)**				27 (17.3%)	15 (15.0%)		28 (17.9%)	12 (12.0%)		25 (16.0%)	14 (14.0%)		24 (15.4%)	14 (14.0%)	
**Unchanged, n (%)**				93 (59.6%)	61 (61.0%)		86 (55.1%)	55 (55.0%)		71 (45.5%)	55 (55.0%)		81 (51.9%)	53 (53.0%)	
**Impaired, n (%)**				36 (23.1%)	24 (24.0%)		42 (26.9%)	33 (33.0%)		60 (38.5%)	31 (31.0%)		51 (32.7%)	33 (33.0%)	
**BN20 Headaches**															
**mean (SD)**	23.1 (26.9)	27.0 (27.5)	0.22	26.7 (27.4)	30.4 (27.8)	0.26	23.9 (26.4)	31.4 (30.7)	0.064	26.1 (28.2)	31.7 (31.9)	0.20	23.5 (28.1)	31.0 (33.3)	0.097
**Change** **mean (SD)**				3.63 (23.82) p = 0.058	3.67 (21.13) p = 0.086	0.80	0.855 (24.814) p = 0.73	4.67 (25.96) p = 0.076	0.21	2.99 (22.20) p = 0.094	4.67 (24.17) p = 0.056	0.56	0.427 (23.642) p = 0.78	4.00 (24.29) p = 0.098	0.22
**Improved, n (%)**				23 (14.7%)	13 (13.0%)		29 (18.6%)	15 (15.0%)		23 (14.7%)	15 (15.0%)		30 (19.2%)	17 (17.0%)	
**Unchanged, n (%)**				96 (61.5%)	62 (62.0%)		96 (61.5%)	59 (59.0%)		98 (62.8%)	58 (58.0%)		99 (63.5%)	58 (58.0%)	
**Impaired, n (%)**				37 (23.7%)	25 (25.0%)		31 (19.9%)	26 (26.0%)		35 (22.4%)	27 (27.0%)		27 (17.3%)	25 (25.0%)	
**BN20 Seizures**															
**mean (SD)**	5.77 (18.22)	0.667 (4.690)	0.0053	5.34 (17.14)	0.654 (4.644)	0.0099	4.49 (14.71)	1.31 (8.02)	0.029	5.98 (16.71)	2.94 (12.50)	0.092	4.06 (12.18)	1.96 (9.17)	0.095
**Change** **mean (SD)**				−0.427 (21.415) p = 0.94	−0.333 (5.793) p = 1.00	0.95	−1.28 (17.30) p = 0.40	0.333 (7.484) p = 1.00	0.98	0.214 (18.741) p = 0.72	2.00 (13.25) p = 0.23	0.76	−1.71 (17.27) p = 0.24	1.000 (5.715) p = 0.25	0.17
**Improved, n (%)**				14 (9.0%)	2 (2.0%)		10 (6.4%)	1 (1.0%)		10 (6.4%)	2 (2.0%)		13 (8.3%)	0 (0.0%)	
**Unchanged, n (%)**				129 (82.7%)	97 (97.0%)		136 (87.2%)	98 (98.0%)		132 (84.6%)	92 (92.0%)		134 (85.9%)	97 (97.0%)	
**Impaired, n (%)**				13 (8.3%)	1 (1.0%)		10 (6.4%)	1 (1.0%)		14 (9.0%)	6 (6.0%)		9 (5.8%)	3 (3.0%)	
**BN20 Drowsiness**															
**mean (SD)**	31.6 (26.7)	32.0 (26.8)	0.87	34.2 (27.3)	34.6 (27.7)	0.95	40.4 (30.5)	43.5 (30.7)	0.46	42.1 (31.2)	40.8 (30.4)	0.71	41.7 (28.7)	41.8 (30.6)	0.81
**Change** **mean (SD)**				2.56 (24.70) p = 0.20	2.67 (20.48) p = 0.20	0.85	8.76 (27.59) p = 0.0001	11.7 (25.2) p < 0.0001	0.57	10.5 (27.5) p < 0.0001	9.00 (24.09) p = 0.0002	0.72	10.0 (27.4) p < 0.0001	10.00 (26.59) p = 0.0002	0.90
**Improved, n (%)**				25 (16.0%)	15 (15.0%)		20 (12.8%)	9 (9.0%)		17 (10.9%)	11 (11.0%)		20 (12.8%)	10 (10.0%)	
**Unchanged, n (%)**				97 (62.2%)	62 (62.0%)		81 (51.9%)	55 (55.0%)		85 (54.5%)	56 (56.0%)		76 (48.7%)	55 (55.0%)	
**Impaired, n (%)**				34 (21.8%)	23 (23.0%)		55 (35.3%)	36 (36.0%)		54 (34.6%)	33 (33.0%)		60 (38.5%)	35 (35.0%)	
**BN20 Itchy skin**															
**mean (SD)**	6.84 (18.05)	5.00 (15.98)	0.28	18.4 (25.5)	10.5 (20.4)	0.0044	17.7 (24.4)	11.1 (24.1)	0.0039	12.4 (21.2)	9.15 (21.06)	0.093	11.3 (20.2)	8.82 (19.86)	0.21
**Change** **mean (SD)**				11.5 (25.0) p < 0.0001	5.67 (17.76) p = 0.0024	0.040	10.9 (23.4) p < 0.0001	6.33 (24.93) p = 0.016	0.039	5.56 (25.35) p = 0.0029	4.00 (23.82) p = 0.11	0.28	4.49 (23.97) p = 0.0085	3.67 (24.57) p = 0.18	0.43
**Improved, n (%)**				11 (7.1%)	2 (2.0%)		10 (6.4%)	4 (4.0%)		13 (8.3%)	7 (7.0%)		13 (8.3%)	8 (8.0%)	
**Unchanged, n (%)**				92 (59.0%)	81 (81.0%)		96 (61.5%)	80 (80.0%)		108 (69.2%)	79 (79.0%)		109 (69.9%)	76 (76.0%)	
**Impaired, n (%)**				53 (34.0%)	17 (17.0%)		50 (32.1%)	16 (16.0%)		35 (22.4%)	14 (14.0%)		34 (21.8%)	16 (16.0%)	
**BN20 Hair loss**															
**mean (SD)**	5.13 (17.00)	3.33 (13.81)	0.30	37.0 (34.2)	11.1 (25.4)	<0.0001	37.4 (32.6)	16.7 (29.6)	<0.0001	28.8 (32.1)	15.4 (26.4)	0.0002	22.6 (31.0)	15.0 (26.8)	0.033
**Change** **mean (SD)**				31.8 (37.4) p < 0.0001	7.00 (19.70) p = 0.0003	<0.0001	32.3 (36.2) p < 0.0001	12.7 (25.0) p < 0.0001	<0.0001	23.7 (31.9) p < 0.0001	11.3 (22.3) p < 0.0001	0.0007	17.5 (32.5) p < 0.0001	11.0 (22.7) p < 0.0001	0.092
**Improved, n (%)**				4 (2.6%)	1 (1.0%)		6 (3.8%)	1 (1.0%)		5 (3.2%)	2 (2.0%)		7 (4.5%)	3 (3.0%)	
**Unchanged, n (%)**				64 (41.0%)	84 (84.0%)		49 (31.4%)	72 (72.0%)		73 (46.8%)	69 (69.0%)		89 (57.1%)	69 (69.0%)	
**Impaired, n (%)**				88 (56.4%)	15 (15.0%)		101 (64.7%)	27 (27.0%)		78 (50.0%)	29 (29.0%)		60 (38.5%)	28 (28.0%)	
**BN20 Weakness of legs**															
**mean (SD)**	5.98 (18.74)	6.67 (17.08)	0.45	8.97 (22.51)	10.8 (22.6)	0.32	9.40 (21.35)	9.48 (21.17)	0.97	10.3 (22.6)	13.1 (25.3)	0.36	9.40 (21.35)	12.1 (21.4)	0.16
**Change** **mean (SD)**				2.99 (17.91) p = 0.042	3.67 (18.89) p = 0.074	0.66	3.42 (14.26) p = 0.0018	2.33 (21.32) p = 0.32	0.45	4.27 (16.81) p = 0.0013	6.00 (27.37) p = 0.036	0.99	3.42 (19.74) p = 0.018	5.00 (21.90) p = 0.024	0.84
**Improved, n (%)**				9 (5.8%)	5 (5.0%)		6 (3.8%)	8 (8.0%)		5 (3.2%)	8 (8.0%)		10 (6.4%)	7 (7.0%)	
**Unchanged, n (%)**				129 (82.7%)	82 (82.0%)		129 (82.7%)	79 (79.0%)		126 (80.8%)	73 (73.0%)		120 (76.9%)	75 (75.0%)	
**Impaired, n (%)**				18 (11.5%)	13 (13.0%)		21 (13.5%)	13 (13.0%)		25 (16.0%)	19 (19.0%)		26 (16.7%)	18 (18.0%)	
**BN20 Bladder control**															
**mean (SD)**	5.56 (15.07)	5.00 (14.51)	0.66	8.76 (21.12)	4.25 (14.59)	0.064	8.76 (20.78)	6.21 (17.38)	0.35	9.19 (21.94)	7.19 (18.54)	0.63	10.0 (24.1)	7.84 (17.66)	0.89
**Change** **mean (SD)**				3.21 (18.07) p = 0.036	−1.000 (12.037) p = 0.58	0.051	3.21 (18.46) p = 0.028	1.00 (16.04) p = 0.51	0.31	3.63 (19.88) p = 0.020	1.67 (16.67) p = 0.42	0.74	4.49 (20.07) p = 0.0041	2.33 (14.41) p = 0.17	0.52
**Improved, n (%)**				5 (3.2%)	8 (8.0%)		9 (5.8%)	8 (8.0%)		9 (5.8%)	7 (7.0%)		9 (5.8%)	5 (5.0%)	
**Unchanged, n (%)**				137 (87.8%)	87 (87.0%)		128 (82.1%)	83 (83.0%)		128 (82.1%)	81 (81.0%)		124 (79.5%)	84 (84.0%)	
**Impaired, n (%)**				14 (9.0%)	5 (5.0%)		19 (12.2%)	9 (9.0%)		19 (12.2%)	12 (12.0%)		23 (14.7%)	11 (11.0%)	
**HAD Anxiety**															
**mean (SD)**	8.25 (3.60)	7.01 (3.36)	0.0015	7.53 (3.67)	5.96 (3.75)	0.0004	7.64 (3.66)	5.92 (3.88)	0.0002	7.66 (3.68)	5.91 (3.97)	0.0004	7.46 (3.78)	6.42 (4.65)	0.055
**0**–**7, n (%)**	61 (38.4%)	55 (52.4%)		78 (49.1%)	70 (66.0%)		68 (42.8%)	73 (68.9%)		74 (46.5%)	69 (65.1%)		75 (47.2%)	59 (55.7%)	
**8**–**10, n (%)**	54 (34.0%)	37 (35.2%)		49 (30.8%)	24 (22.6%)		57 (35.8%)	18 (17.0%)		52 (32.7%)	20 (18.9%)		49 (30.8%)	27 (25.5%)	
**11**–**21, n (%)**	44 (27.7%)	13 (12.4%)	0.0028	32 (20.1%)	12 (11.3%)	0.0066	34 (21.4%)	15 (14.2%)	0.0006	33 (20.8%)	17 (16.0%)	0.017	35 (22.0%)	20 (18.9%)	0.24
**Change, mean (SD)**				−0.717 (2.938) p = 0.0026	−1.000 (2.717) p = 0.0004	0.58	−0.616 (2.985) p = 0.0054	−1.07 (2.91) p = 0.0003	0.33	−0.591 (3.336) p = 0.042	−1.05 (3.23) p = 0.0006	0.23	−0.792 (3.506) p = 0.0067	−0.533 (3.624) p = 0.18	0.46
**Improved, n (%)**				62 (39.0%)	39 (37.1%)		60 (37.7%)	42 (40.0%)		55 (34.6%)	42 (40.0%)		67 (42.1%)	38 (36.2%)	
**Unchanged, n (%)**				62 (39.0%)	49 (46.7%)		64 (40.3%)	46 (43.8%)		64 (40.3%)	43 (41.0%)		50 (31.4%)	38 (36.2%)	
**Impaired, n (%)**				35 (22.0%)	17 (16.2%)		35 (22.0%)	17 (16.2%)		40 (25.2%)	20 (19.0%)		42 (26.4%)	29 (27.6%)	
**HAD Depression**															
**mean (SD)**	9.49 (5.08)	7.72 (5.41)	0.0071	9.30 (5.29)	7.71 (5.59)	0.022	9.52 (4.96)	7.63 (5.59)	0.013	9.70 (4.94)	7.42 (5.34)	0.0004	9.19 (4.99)	7.17 (5.16)	0.0013
**0**–**7, n (%)**	52 (32.7%)	48 (45.3%)		55 (34.6%)	49 (46.2%)		47 (29.6%)	48 (45.3%)		48 (30.2%)	46 (43.4%)		59 (37.1%)	55 (51.9%)	
**8**–**10, n (%)**	20 (12.6%)	14 (13.2%)		17 (10.7%)	12 (11.3%)		28 (17.6%)	14 (13.2%)		26 (16.4%)	21 (19.8%)		17 (10.7%)	12 (11.3%)	
**11**–**21, n (%)**	87 (54.7%)	44 (41.5%)	0.027	87 (54.7%)	45 (42.5%)	0.042	84 (52.8%)	44 (41.5%)	0.018	85 (53.5%)	39 (36.8%)	0.0082	83 (52.2%)	39 (36.8%)	0.011
**Change, mean (SD)**				−0.189 (2.416) p = 0.36	−0.009 (2.520) p = 0.55	0.83	0.025 (2.373) p = 0.92	−0.085 (2.481) p = 0.52	0.93	0.214 (3.258) p = 0.21	−0.302 (3.049) p = 0.21	0.100	−0.302 (3.714) p = 0.94	−0.547 (4.127) p = 0.33	0.54
**Improved, n (%)**				35 (22.0%)	25 (23.6%)		37 (23.3%)	25 (23.6%)		35 (22.0%)	35 (33.0%)		42 (26.4%)	34 (32.1%)	
**Unchanged, n (%)**				96 (60.4%)	62 (58.5%)		87 (54.7%)	62 (58.5%)		82 (51.6%)	44 (41.5%)		71 (44.7%)	43 (40.6%)	
**Impaired, n (%)**				28 (17.6%)	19 (17.9%)		35 (22.0%)	19 (17.9%)		42 (26.4%)	27 (25.5%)		46 (28.9%)	29 (27.4%)	
**ISI**															
**mean (SD)**	4.59 (6.30)	5.75 (7.49)	0.28	5.04 (6.78)	5.56 (7.39)	0.65	5.37 (7.11)	6.42 (8.59)	0.58	5.07 (6.90)	5.78 (7.92)	0.59	3.86 (6.31)	4.54 (7.12)	0.48
**0**–**7**	102 (64.6%)	69 (65.1%)		103 (64.8%)	70 (66.0%)		102 (64.2%)	66 (62.3%)		103 (64.8%)	71 (67.0%)		117 (73.6%)	75 (70.8%)	
**8**–**14**	42 (26.6%)	21 (19.8%)		38 (23.9%)	23 (21.7%)		32 (20.1%)	18 (17.0%)		34 (21.4%)	16 (15.1%)		26 (16.4%)	15 (14.2%)	
**15**–**21**	12 (7.6%)	13 (12.3%)		15 (9.4%)	8 (7.5%)		24 (15.1%)	13 (12.3%)		21 (13.2%)	14 (13.2%)		15 (9.4%)	13 (12.3%)	
**22**–**28**	2 (1.3%)	3 (2.8%)	0.44	3 (1.9%)	5 (4.7%)	0.80	1 (0.6%)	9 (8.5%)	0.17	1 (0.6%)	5 (4.7%)	0.56	1 (0.6%)	3 (2.8%)	0.28
**Change, mean (SD)**				0.475 (5.182) p = 0.087	−0.189 (4.841) p = 0.83	0.19	0.810 (5.897) p = 0.040	0.670 (6.889) p = 0.57	0.22	0.506 (5.626) p = 0.23	0.038 (6.207) p = 0.98	0.35	−0.715 (5.840) p = 0.11	−1.21 (6.35) p = 0.029	0.22
**Improved, n (%)**				13 (8.2%)	11 (10.4%)		14 (8.9%)	14 (13.2%)		16 (10.1%)	15 (14.2%)		27 (17.1%)	22 (20.8%)	
**Unchanged, n (%)**				127 (80.4%)	85 (80.2%)		122 (77.2%)	76 (71.7%)		121 (76.6%)	76 (71.7%)		115 (72.8%)	75 (70.8%)	
**Impaired, n (%)**				18 (11.4%)	10 (9.4%)		22 (13.9%)	16 (15.1%)		21 (13.3%)	15 (14.2%)		16 (10.1%)	9 (8.5%)	
**MFI General Fatigue**															
**mean (SD)**	12.1 (4.5)	12.0 (4.1)	0.89	12.7 (4.5)	12.8 (4.7)	0.69	13.3 (4.5)	13.8 (4.8)	0.26	13.4 (4.3)	13.9 (4.9)	0.19	13.5 (4.3)	13.1 (4.6)	0.53
**Change** **mean (SD)**				0.522 (2.716) p = 0.021	0.802 (3.331) p = 0.014	0.43	1.13 (3.26) p < 0.0001	1.79 (3.62) p < 0.0001	0.11	1.25 (3.53) p < 0.0001	1.88 (3.97) p < 0.0001	0.11	1.37 (3.80) p < 0.0001	1.02 (4.03) p = 0.0090	0.60
**Improved, n (%)**				30 (18.9%)	25 (23.6%)		24 (15.1%)	19 (17.9%)		25 (15.7%)	21 (19.8%)		27 (17.0%)	25 (23.6%)	
**Unchanged, n (%)**				81 (50.9%)	40 (37.7%)		66 (41.5%)	31 (29.2%)		70 (44.0%)	30 (28.3%)		68 (42.8%)	32 (30.2%)	
**Impaired, n (%)**				48 (30.2%)	41 (38.7%)		69 (43.4%)	56 (52.8%)		64 (40.3%)	55 (51.9%)		64 (40.3%)	49 (46.2%)	
**MFI Physical Fatigue**															
**mean (SD)**	11.1 (4.7)	10.6 (4.5)	0.50	11.5 (4.9)	11.3 (4.8)	0.78	12.2 (5.0)	12.2 (5.0)	0.99	12.4 (4.9)	12.3 (5.2)	0.99	12.4 (4.9)	11.7 (4.8)	0.29
**Change** **mean (SD)**				0.428 (2.887) p = 0.076	0.726 (2.984) p = 0.021	0.47	1.13 (3.71) p < 0.0001	1.58 (3.36) p < 0.0001	0.34	1.31 (3.93) p < 0.0001	1.73 (3.96) p < 0.0001	0.41	1.32 (4.14) p < 0.0001	1.10 (4.09) p = 0.0031	0.97
**Improved, n (%)**				28 (17.6%)	21 (19.8%)		28 (17.6%)	17 (16.0%)		31 (19.5%)	22 (20.8%)		34 (21.4%)	22 (20.8%)	
**Unchanged, n (%)**				88 (55.3%)	52 (49.1%)		62 (39.0%)	43 (40.6%)		57 (35.8%)	29 (27.4%)		52 (32.7%)	40 (37.7%)	
**Impaired, n (%)**				43 (27.0%)	33 (31.1%)		69 (43.4%)	46 (43.4%)		71 (44.7%)	55 (51.9%)		73 (45.9%)	44 (41.5%)	
**MFI Reduced Activity**															
**mean (SD)**	11.5 (4.4)	11.7 (4.1)	0.62	12.2 (4.4)	12.0 (4.5)	0.94	12.5 (4.5)	12.7 (4.8)	0.58	12.5 (4.7)	12.5 (4.9)	1.00	12.6 (4.7)	12.0 (4.6)	0.32
**Change** **mean (SD)**				0.667 (2.753) p = 0.0028	0.311 (3.235) p = 0.38	0.29	0.975 (3.328) p = 0.0004	1.01 (3.72) p = 0.0030	0.60	1.01 (3.41) p = 0.0002	0.792 (3.824) p = 0.032	0.53	1.04 (3.52) p = 0.0002	0.255 (3.911) p = 0.51	0.054
**Improved, n (%)**				28 (17.6%)	29 (27.4%)		27 (17.0%)	23 (21.7%)		28 (17.6%)	23 (21.7%)		31 (19.5%)	30 (28.3%)	
**Unchanged, n (%)**				82 (51.6%)	42 (39.6%)		74 (46.5%)	35 (33.0%)		64 (40.3%)	40 (37.7%)		63 (39.6%)	41 (38.7%)	
**Impaired, n (%)**				49 (30.8%)	35 (33.0%)		58 (36.5%)	48 (45.3%)		67 (42.1%)	43 (40.6%)		65 (40.9%)	35 (33.0%)	
**MFI Reduced Motiviation**															
**mean (SD)**	9.27 (3.86)	9.66 (3.65)	0.32	9.63 (3.87)	9.70 (3.66)	0.72	9.92 (4.02)	10.3 (4.3)	0.50	10.2 (4.1)	10.1 (4.3)	0.83	9.91 (4.03)	9.91 (4.02)	0.98
**Change** **mean (SD)**				0.358 (2.615) p = 0.087	0.038 (2.221) p = 0.86	0.22	0.648 (3.194) p = 0.014	0.623 (2.779) p = 0.010	0.76	0.937 (3.097) p = 0.0005	0.443 (2.931) p = 0.22	0.21	0.642 (3.526) p = 0.018	0.245 (3.125) p = 0.65	0.31
**Improved, n (%)**				32 (20.1%)	23 (21.7%)		33 (20.8%)	15 (14.2%)		31 (19.5%)	23 (21.7%)		37 (23.3%)	28 (26.4%)	
**Unchanged, n (%)**				87 (54.7%)	63 (59.4%)		75 (47.2%)	55 (51.9%)		69 (43.4%)	54 (50.9%)		67 (42.1%)	52 (49.1%)	
**Impaired, n (%)**				40 (25.2%)	20 (18.9%)		51 (32.1%)	36 (34.0%)		59 (37.1%)	29 (27.4%)		55 (34.6%)	26 (24.5%)	
**MFI Mental Fatigue**															
**mean (SD)**	10.6 (4.4)	10.9 (4.1)	0.52	10.5 (4.1)	10.7 (4.1)	0.71	11.0 (4.3)	11.6 (4.6)	0.26	11.3 (4.3)	11.2 (4.5)	0.94	11.3 (4.3)	11.0 (4.4)	0.58
**Change** **mean (SD)**				−0.126 (2.482) p = 0.52	−0.245 (3.001) p = 0.46	0.98	0.371 (2.937) p = 0.20	0.679 (3.253) p = 0.038	0.32	0.642 (2.869) p = 0.0075	0.274 (3.337) p = 0.49	0.34	0.679 (3.254) p = 0.0077	0.085 (3.787) p = 0.98	0.17
**Improved, n (%)**				34 (21.4%)	33 (31.1%)		36 (22.6%)	26 (24.5%)		32 (20.1%)	28 (26.4%)		32 (20.1%)	32 (30.2%)	
**Unchanged, n (%)**				89 (56.%)	46 (43.4%)		78 (49.1%)	39 (36.8%)		72 (45.3%)	44 (41.5%)		73 (45.9%)	38 (35.8%)	
**Impaired, n (%)**				36 (22.6%)	27 (25.5%)		45 (28.3%)	41 (38.7%)		55 (34.6%)	34 (32.1%)		54 (34.0%)	36 (34.0%)	

For categorical variables n (%) is presented.

For continuous variables Mean (SD) is presented.

For comparison between groups the Mantel-Haenszel Chi Square test was used for ordered categorical variables and the Mann-Whitney *U* test was used for continuous variables.

For comparison within groups the Wilcoxon Signed Rank test was used Changes in clinical significance over time were assessed in QLQ-C30 [28], QLQ-BN20 [29] based on the observed percentages with decrease or increase of at least five points on the respective subscale or for single items at three-month follow-up.

Clinical relevance was calculated in HADS-A and HADS-D with decrease or increase of at least 1.68 and 1.60 respectively [30], MFI 2.0 points [31] and ISI with a 6-point score [32].

**Table 3:** Missing structure: Global Health (QLQC30), Anxiety (HADS), ISI, General Fatigue (MFI), BNFU (BN20).

The MEANS Procedure					
N = 266, Number (%) of missing values per questionnaire					
**Questionnaire**	Baseline	MID	END	1 month	3 months
QLQ-C30	0 (0)	6 (2)	16 (6)	22 (8)	26 (10)
HADS	2 (1)	8 (3)	17 (6)	24 (9)	28 (11)
ISI	2 (1)	8 (3)	19 (7)	25 (9)	32 (12)
MFI	1 (0)	7 (3)	17 (6)	23 (9)	30 (11)
BN20	10 (4)	13 (5)	24 (9)	32 (12)	36 (13)

### HRQoL and symptoms in the benign tumor group

Statistically significant deteriorations was found between baseline and three months post-treatment, and the worst deteriorations were in global health/QOL, fatigue, dyspnea, appetite loss and hair loss. No significantly improvements between baseline and three months post-treatment were found. Neither HADS-A nor HADS-D scores changed significantly, while insomnia scores indicated significant improvement and the MFI general fatigue and physical fatigue dimension scores increased significantly.

### Clinically significant changes in HRQoL

The incidences of clinically relevant (a change ≥5 points) differences in HRQoL including symptom experience scores are shown in Table 2. Increasing fatigue over time was the most common change, reported by 85 (54%) in the malignant group and 54 (51%) in the benign group. In the malignant group, 82 (52%) participants reported clinically significant deterioration in global health/QOL, as did 49 (46%) in the benign group. A total of 41 (26%) in the malignant group and 30 (28%) in the benign group reported unchanged global health/QoL. No clinically differences were found in HADS, ISI and MFI.

### Medical and demographic factors associated with HRQoL in the malignant and the benign tumor groups

The univariate analysis showed that age, sex, education level and chemotherapy were significantly associated with mental fatigue. However, the multivariable analysis indicated that only age (beta −0.04, 95% CI −0.07; −0.01, *p* = 0.011) and female sex (beta 1.06, 95% CI 0.23; 1.89, *p* = 0.012) were associated with worse mental fatigue. The multivariable analysis showed that marital status (living alone) (beta 3.97, 95% CI 0.33; 7.61, p = 0.033) and more comorbidity (SCQ > 4 points) (beta 6.71 95% CI 1.59; 11.8, *p* = 0.011) were significantly associated with worse motor dysfunction. Further, multivariable analysis showed that higher education level (beta −2.89, 95% CI −5.55; −0.23, *p* = 0.033) were significantly associated with better physical functioning. This data is not shown.

## Discussion

This study investigated HRQoL and acute symptom experiences in PBT-treated patients with primary brain tumors during treatment, and in comparison between baseline and three months after end of treatment. The findings were that HRQoL decreased significantly from baseline to three months post-treatment, in the global health/QOL, physical functioning, role functioning and cognitive functioning domains, in the malignant group. In the benign group, global health/QOL decreased significantly between baseline and three months post-treatment. The most frequently reported symptoms were fatigue and depression in both subgroups.

One difference was that 33% with malignant tumors had also been given chemotherapy, which was significantly associated with negative changes in physical functioning, caused by demanding treatment schedules and side effects. This should be included in the interpretation of data between benign and malignant tumors. The same pattern was found by Geovagnoli et al. [14], who investigated HRQoL among patients with brain tumors, and by Scoccianti et al. [33], who found that patients given both RT and chemotherapy experienced significantly more symptoms. We found significant differences between the two groups concerning HRQoL changes between baseline and three months post-treatment. However, participants in the benign group had reported higher global health at baseline.

Several clinically significant differences were found, that can be interpreted differently, depending on the research perspective [34], [35]. In this study, we performed the non-inferiority analyses based on subscale score changes of at least five points, compared to baseline, according to Osoba et al.’s definition [28]. Clinically significant differences were found in global health, cognitive functioning, fatigue, insomnia, appetite loss, constipation, drowsiness and hair loss in the malignant group. In the benign group, clinically significant differences were found in global health, fatigue, dyspnea, appetite loss, diarrhea, visual disorder, drowsiness and hair loss, important knowledge for health professionals, in order to provide relevant support and care, as confirmed by Snyder et al. [36]. Our previous study [37] evaluated the quality of care in relation to HRQoL at a PBT department, finding that better HRQoL correlated with a higher degree of perceived support for experienced symptoms and vice versa. HRQoL data are thus very valuable in everyday clinical practice. This concurs with Taphoorn et al. [38], who found that routine HRQoL measurement in oncology patients improved communication between patients and medical staff, in addition to providing the staff with information.

During the study period, we found that fatigue in particular increased markedly, possible a direct acute effect of the PBT. A review by Taphoorn et al. demonstrated that HRQoL decreased in brain tumor patients suffering from fatigue [38], concurring with a review by Liu et al. [39], reporting that XRT adversely affected HRQoL by leading to a short-term increase in fatigue [15]. It is unclear whether the decrease in functioning scale scores was due to the treatment or the tumor itself. Further, depression is a common complication in patients with primary brain tumors, and often remains [40]. In this study, over 50% in the malignant group and over 40% in the benign group reported, in the HADS, moderate to severe depressive symptoms at baseline that remained three months after treatment. These results are similar to those reported by Bunevicius et al. [41], who investigated patients with primary brain tumors with self-rating depressive symptom scales. If a self-rating instrument such as the EORTC QLQ-C30 is used as the only instrument to measure psychological distress in patients with brain tumors, there is a risk of under-diagnosed depression.

An additional finding was that the symptom experience was at its worst at the end of treatment, but also that it was still significantly worse three months after treatment, compared with baseline levels. This finding is consistent with that of Bitterlich and Vordermark (2017), who analyzed HRQoL in patients with brain tumors before and after conventional RT. Concurring with the literature, we found that participants who underwent PBT experienced a similar degree of symptoms as those treated with XRT during the treatment period and up to three months after the end of treatment. It would have been desirable to have had a comparable group undergoing XRT. This aspect, and long-term follow-up results, will be studied in a forthcoming study.

The main strength in current study is that all data are patient-reported. Another strength is that HRQoL and associated symptoms were reported prospectively over time, which resulted in a thorough analysis. A limitation is that it is possible that the HRQoL appeared to increase due to selection effects. Patients with low HRQoL may only provide data in the initial stages, due to deterioration of their general health, creating sample distortion toward an apparent improvement in mean HRQoL. A further limitation is the lack of information on supportive treatment e.g. corticosteroids, number of grade II and grade III glioma and the type of benign tumor that were included in the study. This type of treatment may impact HRQoL. Additionally, data on tumor location and performance status over time were not available and these variables may impact patients HRQoL.

## Conclusion

In conclusion, this study found both differences and similarities among and between malignant and benign tumor patients. Global health/QOL in patient with brain tumors is very complex and multidimensional and symptoms are related to patient, tumor and treatment factors. It is important to identify aspects of HRQoL that may be affected by treatment. These include both benefits, expected to improve HRQoL, and negative changes such as symptom experience and associated factors. Further research, including long-term follow-up of PBT- related symptoms in patients with primary brain tumors, is required in order to determine whether symptoms can be reduced by optimizing irradiation technique and other radiation parameters.

## Declaration of Competing Interest

The authors declare that they have no known competing financial interests or personal relationships that could have appeared to influence the work reported in this paper.
